# Milestones in genetics of cerebellar ataxias

**DOI:** 10.1007/s10048-021-00656-3

**Published:** 2021-07-05

**Authors:** Magdalena Krygier, Maria Mazurkiewicz-Bełdzińska

**Affiliations:** Department of Developmental Neurology, Medical University of Gdańsk, ul. Dębinki 7 80-952, Gdańsk, Poland

**Keywords:** Ataxia, Next-generation sequencing, Tandem repeat expansions, Conventional variants, Epigenetics, Genome-wide association studies

## Abstract

Cerebellar ataxias (CAs) comprise a group of rare, neurological disorders characterized by extensive phenotypic and genetic heterogeneity. The core clinical feature is the cerebellar syndrome, which is often accompanied by other neurological or non-neurological signs. In the last 30 years, our understanding of the CA etiology has increased significantly, and numerous ataxia-associated genes have been discovered. Conventional variants or tandem repeat expansions, localized in the coding or non-coding DNA sequences, lead to hereditary ataxia, which can display different patterns of inheritance. Advances in molecular techniques have enabled a rapid and cost-effective detection of causative variants in a significant number of CA patients. However, despite performing extensive investigations, a definite diagnosis is still unknown in the majority of affected individuals. In this review, we discuss the major advances in the genetics of CAs over the last 30 years, focusing on the impact of next-generation sequencing on the genetic landscape of childhood- and adult-onset CAs. Additionally, we outline possible directions for further genetic research in hereditary and sporadic CAs in the era of increasing application of whole-genome sequencing and genome-wide association studies in various neurological disorders.

## Introduction

Cerebellar ataxias (CAs) are a heterogeneous group of neurological disorders characterized by impaired coordination of limb and eye movements, and dysarthria. The primary pathology in CAs is progressive cerebellar atrophy; however, in most cases, the phenotype is complex and involves multiple neurological deficits. Due to large phenotypic and genetic heterogeneity the diagnostic work-up in CAs remains a challenge. In terms of etiology, we distinguish acquired, sporadic, and hereditary ataxias. Hereditary ataxias can be further divided into autosomal dominant cerebellar ataxias (ADCAs, also known as spinocerebellar ataxias, SCAs), autosomal recessive cerebellar ataxias (ARCAs), and X-linked ataxias. Based on similar clinical features, we also distinguish episodic ataxias (EAs) and spastic ataxias (SPAX). Sporadic ataxia, previously known as idiopathic, is a progressive disorder of the cerebellum of unknown etiology, which is neither acquired nor monogenic [1]. The term sporadic is also commonly used in cases with no relevant family history, but for the purposes of this article, it will be used as defined above.

It is difficult to estimate the frequency of specific etiologies among CA patients due to differences in ethnicity, age, family history, and other various inclusion criteria of the studied groups. For many years, the cause of ataxia was unknown in the majority of affected individuals. Since the 1990s, we have observed significant progress in understanding the causes of cerebellar degeneration, mainly due to the development of novel genetic techniques. In this review paper, we discuss milestones in genetics of CAs and outline possible directions for further genetic studies.

## Cerebellar ataxias caused by tandem repeat expansions

After discovery of the first trinucleotide repeat expansions in fragile X syndrome and spinobulbar muscular atrophy in 1991 [2, 3], the era of identifying tandem repeat disorders (TRDs) has begun. Most TRDs in humans are caused by the expansion of short tandem repeats (STRs) (also known as microsatellite DNA), which consist of 1–6 bp repetitive DNA elements. Polyglutamine diseases, caused by the expansion of trinucleotide CAG repeat, account for the majority of TRDs [4]. Among dominantly inherited CAs, CAG repeat expansions were firstly described in SCA1 in 1993, and subsequently in dentatorubral-pallidoluysian atrophy (DRPLA), SCA3, SCA2, SCA6, SCA7, SCA12, and SCA17 [5]. In recessive ataxias, a GAA intronic repeat mutation was described in Friedreich’s ataxia (FRDA) in 1996 [6]. Up to date, there are at least 16 known repeat expansion ataxias. Pathogenic expansions are localized in coding or/and non-coding DNA sequences and comprise repeated motifs from 3 to 6 bp (Table 1). The most recent discovery was a biallelic intronic AAGGG repeat expansion in *RFC1* gene in cerebellar ataxia, neuropathy, vestibular areflexia syndrome (CANVAS) in 2019 [7].

**Table 1:** Tandem repeat expansion ataxias

Tandem repeat expansion ataxias	Gene	Repeat motif	Region	Characteristic clinical features*
Autosomal dominant				
SCA1	*ATXN1*	CAG	Coding	Spasticity, peripheral neuropathy, cognitive decline
SCA2	*ATXN2*	CAG	Coding	Slow saccadic eye movements, peripheral neuropathy, cognitive decline, dopamine-responsive parkinsonism, dystonia, chorea
SCA3	*ATXN3*	CAG	Coding	Facial-lingual fasciculations, pyramidal signs, parkinsonism, peripheral neuropathy, distal muscular atrophy, bulging eyes, autonomic symptoms
SCA6	*CACNA1A*	CAG	Coding	Late-onset, slow progression, diplopia, abnormal vestibuloocular reflex
SCA7	*ATXN7*	CAG	Coding	Cone-rod retinal dystrophy, vision loss, highly variable age of onset and rate of progression
SCA8	*ATXN8OS* and *ATXN8*	CTG and CAG	Non-coding (CTG), coding (CAG)	Scanning dysarthria with characteristic drawn-out slowness of speech, truncal titubation, slow progression
SCA10	*ATXN10*	ATTCT	Non-coding	Recurrent seizures, slow progression
SCA12	*PPP2R2B*	CAG	Non-coding	Upper extremity action tremor, head tremor, subtle parkinsonism, cognitive and psychiatric manifestations
SCA17	*TBP*	CAG and CAA	Coding	Dementia, psychiatric symptoms, chorea, dystonia
SCA31	*BEAN1*	TAAAA, TAGAA, and TGGAA	Non-coding	Late adult-onset, slow progression, normal sensation
SCA36	*NOP56*	GGCCTG	Non-coding	Sensorineural hearing loss, tongue fasciculation and atrophy, upper and lower motor neuron involvement
SCA37	*DAB1*	ATTTC	Non-coding	Dysarthria and abnormal vertical eye movements in early stages of the disease
DRPLA	*ATN1*	CAG	Coding	Choreoathetosis, dementia, seizures, myoclonus, psychiatric symptoms
Autosomal recessive				
Friedreich ataxia	*FXN*	GAA	Non-coding	Sensory axonal neuropathy, absent lower limb tendon reflexes, scoliosis, hypertrophic cardiomyopathy, pes cavus, diabetes mellitus
CANVAS	*RFC1*	AAGGG and AAAGG	Non-coding	Sensory neuropathy or neuronopathy, bilateral vestibular areflexia, chronic cough, autonomic dysfunction
X-linked				
FXTAS	*FMR1*	CGG	Non-coding	Late adult-onset, intention tremor, cognitive decline

*SCA* spinocerebellar ataxia, *DRPLA* dentatorubral-pallidoluysian atrophy, *CANVAS* cerebellar ataxia, neuropathy, vestibular areflexia syndrome; *FXTAS* fragile X-associated tremor/ataxia syndrome; *all have cerebellar ataxia (Web resources: Bird, T.D. (2018). Hereditary Ataxia Overview. In GeneReviews, https://www.ncbi.nlm.nih.gov/books/NBK1138/; OMIM, https://www.omim.org/)

Studies on epidemiology of TRDs show that they are a frequent cause of CA worldwide. FRDA is the most common inherited ataxia in Europe, with the prevalence of 2–4 per 100 000 [8]. Fragile X-associated tremor/ataxia syndrome (FXTAS), caused by a trinucleotide CGG expansion in the *FMR1* gene, is found in 2 to 4% of men with adult-onset CA and a negative family history [9]. A homozygous *RFC1* pentanucleotide expansion, which can manifest not only as CANVAS but also as limited peripheral, vestibular, or cerebellar dysfunction, was detected in 22% of late-onset ataxia cases in the original study [7]. The overall prevalence of SCAs is estimated at 1–3:100 000. The most common SCA worldwide is SCA3, followed by SCA1, SCA2, SCA6, and SCA7 [10]. Polyglutamine SCAs may together account for about half of ADCAs, but there are significant variations in frequency by geographic region [10, 11]. A positive family history highly influences the percentage of detected expansions in the studied groups. While screening for the most common repeat expansion SCAs is routinely performed in patients with CA, the detection rate is rather low in individuals with an informative and negative family history, ranging from 0 to 18.9% [10–14]. However, in some non-familial cases, testing for repeat expansion SCAs should particularly be considered, such as SCA7 in early-onset ataxia and retinal dystrophy (possible dramatic anticipation), SCA6 in late-onset ataxia, and SCA8 in slowly progressive ataxia (possible incomplete penetrance) [10, 14].

## Cerebellar ataxias caused by conventional variants

Since the 1990s, we have observed an increasing number of novel CAs, caused by sequence and copy number variants (CNVs) in various genes. The phenotypes of the most common ARCAs, i.e., ataxia with oculomotor apraxia (AOA) and ataxia-telangiectasia (AT), were differentiated in the 1970s and 1980s [15]. A causative gene for AT was identified in 1995 by positional cloning [16]. In the same year, Ouahchi et al. [17] reported that ataxia with vitamin E deficiency (AVED) is caused by biallelic pathogenic variants in *TTPA* gene. *APTX* and *SETX* gene variants were described as the cause of AOA in 2001 and 2004 respectively [18, 19]. However, it was not until the introduction of next-generation sequencing (NGS) techniques that allowed the identification of multiple novel hereditary cerebellar ataxias, most of which are ultrarare. The application of NGS in clinical practice showed that this method is highly effective in the diagnosis of heterogeneous neurological disorders. From the second decade of the 2000s, many studies have been conducted using NGS in patients with various ataxia-related phenotypes. Three main approaches were used: target sequencing panels, which analyzed the coding exons and flanking introns of a restricted number of genes, whole exome sequencing (WES), and recently whole-genome sequencing (WGS). Prior to NGS, patients underwent numerous diagnostic tests in accordance with the standards of a given center. In general, common repeat expansion CAs had to be excluded by targeted techniques because they are not reliably detected by NGS. In a pilot study Németh et al. [20] analyzed 118 known and candidate ataxia genes in 50 index patients. The overall detection rate was 18% and reached 75% in a subgroup of patients with an adolescent onset and a positive family history. The application of WES in pediatric ataxic patients showed a 46% success rate [21]. Subsequent studies confirmed that the highest percentage of diagnoses can be achieved in groups of early-onset CA, in consanguineous families and in patients with a positive family history. However, such cases represent only a minority of CAs.

## Utility of next-generation sequencing in childhood-onset cerebellar ataxias

Several studies analyzed the utility of NGS in groups of solely children and adolescents with CA. While the overall prevalence of childhood ataxia is relatively high and estimated at 26:100 000 children, a significant proportion can be attributed to acquired and mixed etiology, such as ataxic cerebral palsy (CP) [22]. However, it is a matter of discussion and further investigation of how many of the so-called CP cases are due to a non-progressive brain damage. Hereditary ataxias in children are characterized by large phenotypic and genetic heterogeneity, and ataxia is often a part of a complex phenotype, with multiple comorbidities. Ataxia can be a sign of congenital hindbrain abnormalities (such as Joubert syndrome, Dandy Walker malformation, and pontocerebellar hypoplasia), complex neurodevelopmental disorders (like *MECP2*-disorder), or various metabolic and mitochondrial conditions. Specific non-ataxic symptoms, as well as abnormalities on imaging and laboratory investigations, often guide the diagnosis and enable targeted genetic testing.

Diagnostic rates (DRs) of NGS in pediatric ataxia cohorts vary from 25% to over 80%, depending on the selection of the study group and the type of NGS method (panel vs exome sequencing). Sawyer et al. [21] analyzed the utility of WES in childhood-onset CA and obtained a molecular diagnosis in 13 of 28 families (DR = 46%). Similar result was published by Ohba et al. [23], who evaluated children with cerebellar atrophy and reported a 39% success rate (9 of 28 families). Application of targeted ataxia gene panel in a group of 84 pediatric patients resulted in genetic diagnosis in 25% [24]. In consanguineous families, exome sequencing can provide a molecular diagnosis in up to 80% of cases [25, 26]. Several genes were recurrently implicated in congenital or infantile-onset CAs with cognitive impairment in few series. These are predominantly ion channel-coding genes, such as *CACNA1A, CACNA1G*, *KCNC3*, and *ITPR1*, as well as β-III spectrin gene *SPTBN2*, which is involved in trafficking and stabilization of membrane proteins [25, 27–32]. All are associated with variable degenerative and developmental neurological disorders, including non-progressive or slowly progressive cerebellar ataxia, with a wide range of disease onset, from infancy to adulthood.

More information on the utility of NGS in early-onset cerebellar ataxias (EOCAs) comes from studies on larger series of adults, whose symptoms started appearing before the age of 40. Overall, they showed a diagnostic yield of NGS ranging from 21% to over 50%, with higher percentages of molecular diagnoses in patients with a positive family history compatible with Mendelian inheritance [20, 32–39]. The results of NGS studies outlined the most common etiologies of EOCAs in different populations. According to the literature, the most common recessive ataxias in Western countries are FRDA, spastic paraplegia 7 (SPG7), autosomal recessive spastic ataxia of Charlevoix-Saguenay (ARSACS), AOA2, spectrin repeat-containing nuclear envelope protein type 1 (*SYNE1*)-related ataxia, ataxia-telangiectasia (AT), AOA1, and polymerase gamma (*POLG*)-related ataxia. In addition, Marinesco-Sjögren syndrome and AVED show a relatively high frequency irrespective of ethnic origins, the latter being particularly common in North Africa and in the Mediterranean [40, 41]. All together, they constitute the majority of the more than 100 ARCA etiologies known so far. While most present in childhood and adolescence, onset in adulthood is also frequently described, especially for SPG7 and *SYNE1* ataxias, which are typically adult-onset disorders. In 2019, the International Parkinson and Movement Disorder Society Task Force on Classification and Nomenclature of Genetic Movement Disorders proposed a revised naming system for ARCAs, based on a phenotypical prefix followed by the gene name. Overall, 62 disorders with CA as a prominent feature were assigned with ATX prefix while 30 disorders with CA and coexisting other predominant movement disorder with double prefix [40]. In the same year, the Consensus Statement from the Society for Research on the Cerebellum and Ataxias Task Force proposed a list of 59 primary ARCAs [41]. Furthermore, both classifications listed numerous other disorders that may present with ataxia as an additional feature.

## Utility of next-generation sequencing in adult-onset cerebellar ataxias

Before the era of NGS, genetic diagnosis of SCAs was based mainly on the exclusion of common CAG trinucleotide expansions, which allowed for the diagnosis of approximately half of ADCAs [10, 11, 13]. Advances in molecular techniques have led to identification of numerous novel SCAs, with 48 SCA subtypes and 36 casual genes identified so far [42]. Coutelier et al. [27] examined a large cohort of 412 index cases with dominantly inherited CAs, who tested negative for polyglutamine SCAs, using combining panel sequencing and TaqMan^Õ^ polymerase chain reaction assay and reported a high incidence of channelopathies. Pathogenic variants in *CACNA1A* were found to be the most frequent genetic cause of SCAs in this group, followed by other ion channel-coding genes, such as *KCND3*, *KCNC3*, and *KCNA1.* However, despite a positive first-degree familial history, relevant genetic variants were detected in 15% cases. Similarly, a low diagnostic rate (9.8%) in dominant CAs, negative for CAG repeat expansions, was reported by Chen et al., in the largest sample described so far in China [11]. In this study, over 80% of 480 cases with negative family history remained without a genetic diagnosis. These results indicate that a significant proportion of CAs are genetically unexplained despite strong indicates of genetic contribution. Of note, channelopathies are also a frequent cause of cerebellar ataxia in Canada, but appear to be ultrarare in China and Japan [11, 32, 43].

The majority of patients presenting to ataxia clinics have late onset of symptoms and a negative family history. After excluding acquired and hereditary causes, they are classified as sporadic adult-onset ataxias (SAOAs) [1]. According to the literature, NGS methods may detect conventional variants in 6–33% of apparently SAOA [44–48]. Giordano et al. [48] screened a large cohort of 194 cases with progressive SAOA for causative variants in 201 ataxia-associated genes and obtained a genetic diagnosis in 6%. In a study by Coutelier et al. [34], a diagnostic yield of exome-targeted capture sequencing in patients with disease onset after 40 years of age was 6.4%. Klockgether and Giordano et al. [1, 48] estimated that testing for common tandem repeat expansions, followed by ataxia-specific NGS panel, may result in genetic diagnosis in about 20% of apparently SAOAs. Apart from detecting ultrarare monogenic causes, NGS studies outlined several genes commonly implicated in adult-onset CAs, such as *SYNE1*, *SPG7*, and *ANO10* [34, 36, 44]. Importantly, a significant number of patients classified as CAs were found to carry pathogenic variants traditionally associated with hereditary spastic paraplegias (HSPs). This indicates that ataxias and HSPs share similar pathways and mechanisms and gave rise to the concept of a continuous ataxia-spasticity disease spectrum [49]. Table 2 presents genes recurrently involved in several series of ataxic patients and reported as common causes of rare ataxias.

**Table 2:** Genes recurrently involved in several series of ataxic patients, with pathogenic variants detected by next-generation sequencing

Gene	Genetic nomenclature*	Cerebellar ataxia-related phenotypes (OMIM)	Inheritance	Characteristic clinical features**	Age at onset	References
*CACNA1A*	-	Episodic ataxia type 2	Autosomal dominant	High phenotypic heterogeneity of loss-of-function mutations, including: episodic and/or progressive cerebellar ataxia, cognitive impairment, hemiplegic migraine, epileptic encephalopathy, autism, paroxysmal non-epileptic events	From infancy to adulthood	[23, 25, 27, 32, 34, 36, 47, 48, 51]
		Familial hemiplegic migraine type 1 with progressive cerebellar ataxia				
		Spinocerebellar ataxia type 6				
*ITPR1*	-	Spinocerebellar ataxia 15	Autosomal dominant	Slow progression, titubation, upper limb postural tremor	Mean 35 years (range 18–66 years)	[23, 25, 27, 29, 32, 46, 47]
		Spinocerebellar ataxia 29, congenital non-progressive		Slow progression or non-progressive, cognitive deficits	Onset at birth	
	ATX-ITPR1	Gillespie syndrome	Autosomal dominant and autosomal recessive	Partial aniridia, hypotonia, intellectual disability	Onset at birth	
*SPTBN2*	-	Spinocerebellar ataxia 5	Autosomal dominant	Slow progression, intention tremor, mild facial myokymia; heterozygous mutations can also cause congenital non-progressive ataxia with psychomotor delay	Variable (from birth to 50 years)	[27, 28, 51]
	ATX-SPTBN2	Spinocerebellar ataxia, autosomal recessive 14	Autosomal recessive	Slow progression, global developmental delay, cognitive impairment	Onset in infancy	
*SPG7*	HSP/ATX-SPG7	Spastic paraplegia 7	Autosomal recessive (autosomal dominant also reported)	Spastic paraplegia, impaired vibration sense, optic atrophy, ophthalmoplegia	Mean 30 years (range 11–72 years)	[27, 34, 36, 39, 44–48, 51]
*ANO10*	ATX-ANO10	Spinocerebellar ataxia, autosomal recessive 10 (autosomal recessive cerebellar ataxia type 3)	Autosomal recessive	Slow progression, pyramidal signs, bradykinesia, pes cavus, motor neuron involvement, epilepsy, cognitive decline	Typically in adulthood (from teens to between 27 and 53 years)	[36, 44, 47]
*SYNE1*	ATX-SYNE1	Spinocerebellar ataxia, autosomal recessive 8; (autosomal recessive cerebellar ataxia type 1)	Autosomal recessive	Pure cerebellar syndrome, upper and lower motor neuron features, cerebellar cognitive and affective syndrome, possible early-onset multisystemic disease with respiratory dysfunction and intellectual disability	Typically in adulthood (range 6–45 years)	[32, 34, 37, 44–47, 51]
*SACS*	ATX/HSP-SACS	Spastic ataxia, Charlevoix-Saguenay type	Autosomal recessive	Peripheral sensorimotor neuropathy, lower limb spasticity, hypermyelinated retinal fibers	Typically in infancy or early childhood (range 0–40 years)	[20, 21, 24, 25, 32–34, 39]
*SETX*	ATX-SETX	Spinocerebellar ataxia, autosomal recessive, with axonal neuropathy 2 (ataxia with oculomotor apraxia type 2)	Autosomal recessive	Axonal sensorimotor neuropathy, oculomotor apraxia (in about half of individuals), elevated serum concentration of alpha-fetoprotein	Mean 14 years (range 3–30 years)	[20, 21, 24, 32–36]

^*^According to the International Parkinson and Movement Disorder Society [40]; **all have cerebellar ataxia (Web resources: Bird, T.D. (2018). Hereditary Ataxia Overview. In GeneReviews, https://www.ncbi.nlm.nih.gov/books/NBK1138/; OMIM, https://www.omim.org/)

## Future genetic testing in cerebellar ataxias

Despite the wide application of NGS in the diagnosis of heterogeneous neurological diseases, it is well known that these methods have several limitations. Multigene panels have proven to be an effective diagnostic tool, showing significant diagnostic yield at relatively low cost. However, they analyze a limited number of genes, which may pose an important problem in the context of the heterogeneity of CAs. Clinical features can overlap with other neurological disorders and can be separately classified as leukodystrophies, metabolic disorders, spastic paraplegias, and intellectual disability, and therefore causative variants may be not captured by a typical ataxia-specific gene panel. In this respect, WES has the advantage of analyzing the sequence of nearly 95% protein-coding regions in the genome. Recently, due to continuous improvements in NGS and bioinformatics data analysis, WES has become widely available in clinical setting.

In terms of CAs, major limitations of NGS are the problem with detection of tandem repeat expansions and diseases caused by alterations in mitochondrial DNA. WES is also considered not adequate for detecting deep intronic variants, copy number variations (CVNs) defined as single exon or larger deletions and duplications, balanced translocations or complex inversions, and low-level mosaicism. There may also be problems in achieving good coverage of the GC-rich regions of the exome [50]. However, many of these issues have been recently addressed. Now, many services offer multigene panels and exome sequencing together with analysis of mtDNA, CNVs and selected deep intronic variants, with efficient capture and satisfying read depth. Although CNVs constitute a minority of variants in progressive CAs, their involvement cannot be ignored. Ngo et al. [46] performed WES in a heterogeneous cohort of 260 patients with CA and/or spastic paraplegia, with additional CNV and repeat expansion analysis in a representative subset of cases (*n* = 68) and found two pathogenic CNVs and one trinucleotide expansion. In a study by Marelli et al. [35], a group of 33 patients was examined using mini-exome coupled to read depth-based CNV and found two pathogenic CNVs in *SETX* gene. While in autosomal recessive diseases the diagnosis is usually facilitated by the presence of a concomitant SNV in the second allele, undetected CNVs may be a potential cause of false-negative results, especially in cases with de novo dominant variants. The combination of multiple NGS approaches, such as exome sequencing, targeted testing, CNV, and repeat expansion analysis, can provide high diagnostic yield of over 50% in various heterogeneous ataxia cohorts [31, 46, 51].

Among patients with unexplained CA, the use of WGS may be a promising option. WGS is designed to analyze all coding and non-coding sequences of nuclear DNA and covers up to 98% of the whole human genome. By comparison, WES captures up to 95% of the exome, which is only 1–2% of the human genome. Additionally, WGS has more uniform depth of coverage and is more efficient than WES for detecting SNVs, small insertions and deletions (indels), and CNVs within regions that are targeted by WES [52]. Indeed, data show that so far diagnostic advantage of WGS over WES consists mainly in better detection of alterations in the coding regions of the genome [53]. Nevertheless, CAs caused by variants in non-coding DNA sequences have also been reported, like early-onset cerebellar ataxia associated with non-coding RNA, RNU12 [54]. Determination of pathogenicity of deep intronic variants remains a challenge, but this is likely to change in the future with the further improvements in functional studies and bioinformatics tools.

So far, there are only a few studies in the literature assessing the utility of WGS in CAs. Kang et al. [55] performed WGS in patients with CA after negative testing for repeat expansions and multigene panel and found a causative variant in one out of three individuals. Kim et al. [56] examined a heterogeneous group of 18 cases with spastic paraplegia with or without CA and reported a 38.9% diagnostic rate. However, only one intronic variant was detected that could have been missed on WES. Further research on larger groups of patients is necessary to determine the benefits of WGS in CAs.

In the case of CAs, the possibility of detecting tandem repeat expansions using WES and WGS is particularly promising. Standard NGS techniques based on short-read sequencing were traditionally not capable to detect TRDs, except for SCA6, caused by the smallest expansion. Recently, several algorithms were developed to analyze STRs from short-read NGS data and successfully applied in CA patients. Retrospective use of these algorithms can lead to diagnosis in individuals with negative NGS results. Additionally, we can expect to identify new ataxia-causative expansions in the future [57]. Recently, WGS in conjunction with non-parametric linkage analysis has led to the identification of pathogenic repeat expansion in CANVAS and late-onset ataxia [7]. WGS also contributed to the discovery of several novel TRDs, such as benign adult familial myoclonic epilepsy, neuronal intranuclear inclusion disease, oculopharyngodistal myopathy, and others [58, 59]. At present, there are several types of SCA, i.e., SCA4, SCA25, SCA30 and SCA32, which genetic cause awaits to be determined. The introduction of new molecular technologies, such as PacBio and Nanopore long-read sequencing, which enables sequencing normal and expanded STR alleles, can lead to further discoveries in the field of repeat expansion disorders in the future [57].

## Cerebellar ataxias—beyond monogenic diseases

Despite the increasing use of advanced genetic techniques, still more than half of patients with CA remain without a specific diagnosis [60]. Determining the cause of the disease is of particular concern in patients with late-onset of symptoms and a negative family history, who constitute the majority of CAs. The term idiopathic late-onset CA has previously been used for all individuals without an apparent acquired or hereditary etiology. Further studies allowed to distinguish from this group patients with multiple system atrophy of cerebellar type (MSA-C), a separate disease entity characterized by the presence of glial cytoplasmic inclusions. SAOA has to be differentiated from MSA-C, and its cause remains unknown. Despite significant phenotypic heterogeneity, SAOA patients present several common features, such as isolated cerebellar atrophy on neuroimaging, pyramidal signs, absent ankle reflexes, reduced vibration sense, and mild urinary symptoms. The mean age of onset reported in the literature varies from 41 to 56 years, and disease progression is significantly slower than in MSA-C [1].

Presumably some patients with sporadic CA are patients with undiagnosed acquired, autoimmune, or hereditary causes. With the introduction of WGS, we can expect identification of novel monogenic etiologies, especially in early-onset and familial cases. However, it appears that a significant proportion of sporadic ataxias may have multifactorial or polygenic background. Genome-wide association studies (GWASs) identify genomic risk variants by a patient to population control variant frequency comparison. To date, no GWASs have been published in the population of patients with sporadic CA. The main issue is that GWASs require large groups of patients and controls, typically thousands of individuals, which is difficult to achieve in CAs. Moreover, an additional challenge is to distinguish phenotypically homogeneous cases. So far, GWASs for common and complex neurodegenerative disorders, such as Alzheimer’s disease (AD), Parkinson’s disease, amyotrophic lateral sclerosis, and frontotemporal dementia, identified several susceptibility variants, but most of them have very small effect on risk [61]. GWAS in MSA found no significant loci, but detected several potential variants that need to be examined in a larger sample set [62]. Studies show that AD has a significant polygenic component, which may be used in calculating genetic risk of developing the disease. The sum of risk alleles carried by an individual, where each single nucleotide polymorphism (SNP) is weighted by the effect size from the prior GWAS, is called a polygenic risk score (PRS) and may have a predictive value for multiple common diseases. Promising results that point to potential clinical utility of PRS in the future have been published for AD and epilepsy [63, 64]. Collecting a large number of clinically homogeneous ataxia patients through international consortia gives hope to conduct GWASs and identify potential variants enriched in CA.

Epigenetic alterations may potentially be responsible for some cases of unexplained CAs; however, there is little literature on this topic. Epigenetic mechanisms such as DNA methylation, histone modifications, and microRNAs (miRNAs) regulate gene expression without changing the underlying genomic sequence and are implicated in various neurodevelopmental processes. Studies on epigenetics in CAs have shown that epigenetic dysregulation is involved in the pathogenesis of FRDA, FXTAS, ataxia-telangiectasia, and several SCAs [65, 66]. MiRNAs have been shown to be essential for the survival of Purkinje cells, and their loss leads to the degeneration of the cerebellum and the development of ataxia [67]. Alterations in specific miRNAs levels have been described in SCA1 and SCA3. Given the role of miRNAs in CA neuropathology, it was suggested that pathogenic variants in miRNA-binding sites or miRNAs may be causative for a group of unexplained ataxias [66]. Aberrant DNA methylation profiles were found in several trinucleotide expansion disorders and related to age at onset and somatic repeat instability of the mutated allele [68]. Recently, genome-wide DNA methylation profiling showed differentially methylated loci in ataxia-telangiectasia [69]. Further studies are needed to determine the possible role of epigenetic dysregulation in the pathogenesis of unexplained CAs.

## Summary

The last 30 years have been the era of discovering the monogenic causes of CAs. After trinucleotide expansion ataxias and several conventional ataxias, identified by targeted techniques, there was a rapid increase in the number of novel ataxia-causative variants, detected by massive parallel sequencing. The introduction of NGS to clinical and experimental neurology significantly changed our understanding of the complex landscape of cerebellar ataxia genetics. Currently, the common use of multigene panels and WES in clinical practice allow for the rapid diagnosis of CA etiology in a substantial number of patients. However, for the majority of affected individuals, especially adults, genetic findings do not currently inform diagnosis or management. In fact, a significant proportion of CAs remains genetically unexplained despite strong indicates of genetic contribution. Further advances in NGS technique and bioinformatics analysis, as well as the widespread use of WGS, give hope for better detection of conventional variants and repeat expansions in the future. In addition, GWASs will be needed in large series of ataxic patients in order to identify risk loci contributing to cerebellar ataxia. Combining GWASs results with rare-variant burden analyses and repeat expansion data from whole-genome sequencing may unravel the genetic architecture of unexplained CAs.

## Data Availability

Not applicable.

## References

[1] Klockgether T (2018). Sporadic adult-onset ataxia. Handb Clin Neurol.

[2] Verkerk AJ, Pieretti M, Sutcliffe JS (1991). Identification of a gene (FMR-1) containing a CGG repeat coincident with a breakpoint cluster region exhibiting length variation in fragile X syndrome. Cell.

[3] La Spada AR, Wilson EM, Lubahn DB, Harding AE, Fischbeck KH (1991). Androgen receptor gene variants in X-linked spinal and bulbar muscular atrophy. Nature.

[4] Hannan AJ (2018). Tandem repeats mediating genetic plasticity in health and disease. Nat Rev Genet.

[5] Klockgether T, Paulson H (2011). Milestones in ataxia. Mov Disord.

[6] Campuzano V, Montermini L, Moltò MD et al (1996) Friedreich’s ataxia: autosomal recessive disease caused by an intronic GAA triplet repeat expansion. Science 271(5254):1423–1427. 10.1126/science.271.5254.1423 10.1126/science.271.5254.1423 8596916

[7] Cortese A, Simone R, Sullivan R (2019). Biallelic expansion of an intronic repeat in RFC1 is a common cause of late-onset ataxia. Nat Genet.

[8] Bidichandani SI, Delatycki MB (1998) Friedreich Ataxia. In: Adam MP, Ardinger HH, Pagon RA et al (eds) GeneReviews® [Internet]. Seattle (WA): University of Washington, Seattle

[9] Brussino A, Gellera C, Saluto A (2005). FMR1 gene premutation is a frequent genetic cause of late-onset sporadic cerebellar ataxia. Neurology.

[10] Durr A (2010). Autosomal dominant cerebellar ataxias: polyglutamine expansions and beyond. Lancet Neurol.

[11] Chen Z, Wang P, Wang C (2018). Updated frequency analysis of spinocerebellar ataxia in China. Brain.

[12] Wardle M, Majounie E, Muzaimi MB, Williams NM, Morris HR, Robertson NP (2009). The genetic aetiology of late-onset chronic progressive cerebellar ataxia. A population-based study. J Neurol.

[13] Moseley ML, Benzow KA, Schut LJ (1998). Incidence of dominant spinocerebellar and Friedreich triplet repeats among 361 ataxia families. Neurology.

[14] Schöls L, Szymanski S, Peters S (2000). Genetic background of apparently idiopathic sporadic cerebellar ataxia. Hum Genet.

[15] Aicardi J, Barbosa C, Andermann E (1988). Ataxia-ocular motor apraxia: a syndrome mimicking ataxia-telangiectasia. Ann Neurol.

[16] Savitsky K, Bar-Shira A, Gilad S (1995). A single ataxia telangiectasia gene with a product similar to PI-3 kinase. Science.

[17] Ouahchi K, Arita M, Kayden H (1995). Ataxia with isolated vitamin E deficiency is caused by variants in the alpha-tocopherol transfer protein. Nat Genet.

[18] Moreira MC, Klur S, Watanabe M (2004). Senataxin, the ortholog of a yeast RNA helicase, is mutant in ataxia-ocular apraxia 2. Nat Genet.

[19] Date H, Onodera O, Tanaka H (2001). Early-onset ataxia with ocular motor apraxia and hypoalbuminemia is caused by variants in a new HIT superfamily gene. Nat Genet.

[20] Németh AH, Kwasniewska AC, Lise S (2013). Next generation sequencing for molecular diagnosis of neurological disorders using ataxias as a model. Brain.

[21] Sawyer SL, Schwartzentruber J, Beaulieu CL (2014). Exome sequencing as a diagnostic tool for pediatric-onset ataxia. Hum Mutat.

[22] Musselman KE, Stoyanov CT, Marasigan R (2014). Prevalence of ataxia in children: a systematic review. Neurology.

[23] Ohba C, Osaka H, Iai M (2013). Diagnostic utility of whole exome sequencing in patients showing cerebellar and/or vermis atrophy in childhood. Neurogenetics.

[24] Arslan EA, Öncel İ, Ceylan AC, Topçu M, Topaloğlu H (2020). Genetic and phenotypic features of patients with childhood ataxias diagnosed by next-generation sequencing gene panel. Brain Dev.

[25] Valence S, Cochet E, Rougeot C (2019). Exome sequencing in congenital ataxia identifies two new candidate genes and highlights a pathophysiological link between some congenital ataxias and early infantile epileptic encephalopathies. Genet Med.

[26] Jiao B, Zhou Z, Hu Z (2020). Homozygosity mapping and next generation sequencing for the genetic diagnosis of hereditary ataxia and spastic paraplegia in consanguineous families. Parkinsonism Relat Disord.

[27] Coutelier M, Coarelli G, Monin ML (2017). A panel study on patients with dominant cerebellar ataxia highlights the frequency of channelopathies. Brain.

[28] Nicita F, Nardella M, Bellacchio E (2019). Heterozygous missense variants of SPTBN2 are a frequent cause of congenital cerebellar ataxia. Clin Genet.

[29] Synofzik M, Helbig KL, Harmuth F (2018). De novo ITPR1 variants are a recurrent cause of early-onset ataxia, acting via loss of channel function. Eur J Hum Genet.

[30] Travaglini L, Nardella M, Bellacchio E (2017). Missense variants of CACNA1A are a frequent cause of autosomal dominant nonprogressive congenital ataxia. Eur J Paediatr Neurol.

[31] Chemin J, Siquier-Pernet K, Nicouleau M (2018). De novo mutation screening in childhood-onset cerebellar atrophy identifies gain-of-function variants in the CACNA1G calcium channel gene. Brain.

[32] Gauquelin L, Hartley T, Tarnopolsky M (2020). Channelopathies are a frequent cause of genetic ataxias associated with cerebellar atrophy. Mov Disord Clin Pract.

[33] Shakya S, Kumari R, Suroliya V (2019). Whole exome and targeted gene sequencing to detect pathogenic recessive variants in early onset cerebellar ataxia. Clin Genet.

[34] Coutelier M, Hammer MB, Stevanin G (2018). Efficacy of exome-targeted capture sequencing to detect variants in known cerebellar ataxia genes. JAMA Neurol.

[35] Marelli C, Guissart C, Hubsch C (2016). Mini-exome coupled to read-depth based copy number variation analysis in patients with inherited ataxias. Hum Mutat.

[36] Bogdanova-Mihaylova P, Hebert J, Moran S (2021). Inherited cerebellar ataxias: 5-year experience of the Irish National Ataxia Clinic. Cerebellum.

[37] Krygier M, Kwarciany M, Wasilewska K (2019). A study in a Polish ataxia cohort indicates genetic heterogeneity and points to MTCL1 as a novel candidate gene. Clin Genet.

[38] Vural A, Şimşir G, Tekgül Ş (2021). The complex genetic landscape of hereditary ataxias in Turkey and implications in clinical practice. Mov Disord.

[39] Pyle A, Smertenko T, Bargiela D (2015). Exome sequencing in undiagnosed inherited and sporadic ataxias. Brain.

[40] Rossi M, Anheim M, Durr A (2018). The genetic nomenclature of recessive cerebellar ataxias. Mov Disord.

[41] Beaudin M, Matilla-Dueñas A, Soong BW (2019). The classification of autosomal recessive cerebellar ataxias: a consensus statement from the Society for Research on the Cerebellum and Ataxias Task Force. Cerebellum.

[42] Online Mendelian Inheritance in Man, OMIM®. McKusick-Nathans Institute of Genetic Medicine, Johns Hopkins University (Baltimore, MD). World Wide Web URL: https://omim.org/. Accessed 20 May 2021

[43] Tada Y, Kume K, Matsuda Y (2020). Genetic screening for potassium channel variants in Japanese autosomal dominant spinocerebellar ataxia. J Hum Genet.

[44] Keogh MJ, Steele H, Douroudis K (2015). Frequency of rare recessive variants in unexplained late onset cerebellar ataxia. J Neurol.

[45] Fogel BL, Lee H, Deignan JL (2014). Exome sequencing in the clinical diagnosis of sporadic or familial cerebellar ataxia. JAMA Neurol.

[46] Ngo KJ, Rexach JE, Lee H (2020). A diagnostic ceiling for exome sequencing in cerebellar ataxia and related neurological disorders. Hum Mutat.

[47] Kim M, Kim AR, Kim JS (2020). Clarification of undiagnosed ataxia using whole-exome sequencing with clinical implications. Parkinsonism Relat Disord.

[48] Giordano I, Harmuth F, Jacobi H (2017). Clinical and genetic characteristics of sporadic adult-onset degenerative ataxia. Neurology.

[49] Synofzik M, Schüle R (2017). Overcoming the divide between ataxias and spastic paraplegias: Shared phenotypes, genes, and pathways. Mov Disord.

[50] Meienberg J, Zerjavic K, Keller I (2015). New insights into the performance of human whole-exome capture platforms. Nucleic Acids Res.

[51] Sun M, Johnson AK, Nelakuditi V (2019). Targeted exome analysis identifies the genetic basis of disease in over 50% of patients with a wide range of ataxia-related phenotypes. Genet Med.

[52] Belkadi A, Bolze A, Itan Y (2015). Whole-genome sequencing is more powerful than whole-exome sequencing for detecting exome variants. Proc Natl Acad Sci U S A.

[53] Alfares A, Aloraini T, Subaie LA (2018). Whole-genome sequencing offers additional but limited clinical utility compared with reanalysis of whole-exome sequencing. Genet Med.

[54] Elsaid MF, Chalhoub N, Ben-Omran T (2017). Mutation in noncoding RNA RNU12 causes early onset cerebellar ataxia. Ann Neurol.

[55] Kang C, Liang C, Ahmad KE (2019). High degree of genetic heterogeneity for hereditary cerebellar ataxias in Australia. Cerebellum.

[56] Kim A, Kumar KR, Davis RL (2019). Increased diagnostic yield of spastic paraplegia with or without cerebellar ataxia through whole-genome sequencing. Cerebellum.

[57] Bahlo M, Bennett MF, Degorski P, Tankard RM, Delatycki MB, Lockhart PJ (2018). Recent advances in the detection of repeat expansions with short-read next-generation sequencing. F1000Res.

[58] Ishiura H, Shibata S, Yoshimura J (2019). Noncoding CGG repeat expansions in neuronal intranuclear inclusion disease, oculopharyngodistal myopathy and an overlapping disease. Nat Genet.

[59] Ishiura H, Doi K, Mitsui J (2018). Expansions of intronic TTTCA and TTTTA repeats in benign adult familial myoclonic epilepsy. Nat Genet.

[60] Galatolo D, Tessa A, Filla A, Santorelli FM (2018). Clinical application of next generation sequencing in hereditary spinocerebellar ataxia: increasing the diagnostic yield and broadening the ataxia-spasticity spectrum A retrospective analysis. Neurogenetics.

[61] Pihlstrøm L, Wiethoff S, Houlden H (2017). Genetics of neurodegenerative diseases: an overview. Handb Clin Neurol.

[62] Sailer A, Scholz SW, Nalls MA (2016). A genome-wide association study in multiple system atrophy. Neurology.

[63] Escott-Price V, Myers AJ, Huentelman M, Hardy J (2017). Polygenic risk score analysis of pathologically confirmed Alzheimer disease. Ann Neurol.

[64] Leu C, Stevelink R, Smith AW (2019). Polygenic burden in focal and generalized epilepsies. Brain.

[65] Serrano M (2018). Epigenetic cerebellar diseases. Handb Clin Neurol.

[66] Smeets CJ, Verbeek DS (2014). Cerebellar ataxia and functional genomics: identifying the routes to cerebellar neurodegeneration. Biochim Biophys Acta.

[67] Schaefer A, O’Carroll D, Tan CL et al (2007) Cerebellar neurodegeneration in the absence of microRNAs. J Exp Med 204(7):1553–1558. 10.1084/jem.20070823 10.1084/jem.20070823 PMC2118654 17606634

[68] Ding D, Wang C, Chen Z (2019). Polymorphisms in DNA methylation-related genes are linked to the phenotype of Machado-Joseph disease. Neurobiol Aging.

[69] McGrath-Morrow SA, Ndeh R, Helmin KA (2020). DNA methylation and gene expression signatures are associated with ataxia-telangiectasia phenotype. Sci Rep.

